# Alteration of microRNA-4474/4717 expression and CREB-binding protein in human colorectal cancer tissues infected with *Fusobacterium nucleatum*

**DOI:** 10.1371/journal.pone.0215088

**Published:** 2019-04-05

**Authors:** Yu-yang Feng, Dong-zhu Zeng, Ya-nan Tong, Xiao-xue Lu, Guo-dong Dun, Bin Tang, Zhu-jun Zhang, Xin-li Ye, Qian Li, Jian-ping Xie, Xu-hu Mao

**Affiliations:** 1 Institute of Modern Biopharmaceuticals, State Key Laboratory Breeding Base of Eco-Environment and Bio-Resource of the Three Gorges Area, Key Laboratory of Eco-environments in Three Gorges Reservoir Region, Ministry of Education, School of Life Sciences, Southwest University, Chongqing, China; 2 Department of Clinical Microbiology and Immunology, Southwest Hospital & College of Pharmacy and Medical Laboratory Science, Army Medical University (Third Military Medical University), Chongqing, China; 3 Digestive Disease Center, Third Affiliated Hospital of Chongqing Medical University, Chongqing, China; 4 Department of Hospital Infection Control, Southwest Hospital, Army Medical University (Third Military Medical University), Chongqing, China; University of New Mexico, UNITED STATES

## Abstract

Colorectal cancer (CRC) is a common and highly lethal form of cancer. Although the etiologic role of *Fusobacterium nucleatum* (*F*. *nucleatum*) in the development of CRC has been elucidated, the specific tumor molecules involved in the progression of CRC induced by *F*. *nucleatum* have not been identified. This study investigated several miRNAs and genes involved in the progression of *F*. *nucleatum-*induced CRC by Affymetrix miRNA microarray technology and GeneChip Human Transcriptome Array 2.0. The results suggest that miR-4474 and miR-4717 are up-regulated in CRC tissues in response to *F*. *nucleatum* infection, compared with the control group (paracancerous tissues), while other genes associated with signaling pathways in cancer, including CREB-binding protein (CREBBP), STAT1, PRKACB, CAMK2B, JUN, TP53 and EWSR1, were dysregulated. Bioinformatic analysis identified CREBBP as the primary aberrantly expressed gene in *F*. *nucleatum*-induced CRC. Consistent with the microarray analysis results, real-time RT-PCR analysis demonstrated that the expression of miR-4474/4717 was upregulated while that of CREBBP mRNA was downregulated in CRC patients infected with *F*. *nucleatum*. Additionally, CREBBP was identified as a novel target of miR-4474/4717. The results of this study suggest that miR-4474 and miR-4717 are involved in the progression of *F*. *nucleatum-*induced CRC by posttranscriptionally regulating the target gene CREBBP.

## Introduction

Colorectal cancer (CRC) is a common and one of the most lethal cancers ever identified [1], with more than one million new CRC cases are reported annually that result in approximately seven hundred thousand deaths [1]. *Fusobacterium nucleatum* (*F*. *nucleatum*) is a gram-negative anaerobic bacterium associated with periodontitis, appendicitis, Lemierre's disease, inflammatory bowel disease (IBD) and CRC [2,3,4]. Located at the intestinal interface, *F*. *nucleatum* is widely recognized as a risk factor for colorectal carcinogenesis. Previous studies focused on the expression of oncogenes and associated genes in CRC tissues [5,6], while the molecular mechanism of CRC resulting from *F*. *nucleatum* infection has not been fully elucidated. Therefore, it is of great importance to screen and identify the specific tumor molecules involved in the progression of CRC induced by *F*. *nucleatum* to facilitate further studies on the prevention and therapy of CRC.

MicroRNAs (miRNAs) are small noncoding RNA molecules (approximately 19 to 25 nucleotides in length) that negatively regulate the expression of target genes at the posttranscriptional level. MicroRNAs play an important regulatory role in various biological processes and are involved in several biological functions in tumor cells (e.g., migration, proliferation, invasion, and differentiation) [7]. Previous studies have reported that many miRNAs (e.g., miR-21, miR-25 and miR-92) are involved in the development and progression of human CRC as oncogenes or tumor suppressor genes [8,9]. Nevertheless, few studies have investigated the roles of aberrant miRNAs expression induced by *F*. *nucleatum* infection in human CRC.

CREB-binding protein (CREBBP) has been reported to be involved in CRC [6]. CREBBP is a central and key node in eukaryotic transcriptional regulatory networks that interacts with more than four hundred transcription factors and other regulatory proteins [10]. As one of the most important oncogenes, CREBBP plays a crucial role in the regulation of cell growth, metabolism, differentiation, and angiogenesis [11]. In addition, CREBBP promotes CRC initiation via the Wnt/β-catenin signaling pathway [10,11], which is a key pathway in the genesis and development of many malignant human tumors [12,13].

In this study, miR-4474/4717 and CREBBP were demonstrated to be specific miRNAs associated with CRC caused by *F*. *nucleatum* infection by microarray analysis and other experiments. Additionally, miR-4474/4717 and their target gene CREBBP were further investigated to provide new insight into the pathogenic process of CRC caused by *F*. *nucleatum* infection.

## Material and methods

### Materials

Fifteen CRC tissues and ten paracancerous tissues from individuals receiving curative operations during January 2017 and August 2018 at the Southwest Hospital, Chongqing, China, were assayed by microarray. The tissues were collected and immediately frozen in liquid nitrogen until RNA isolation, the paracancerous normal tissues were used as the control groups, and the *F*. *nucleatum* infection status was determined by their 16S rRNA gene copy number and *F*. *nucleatum* culture results. Patients were defined as being *F*. *nucleatum* positive if both tests yielded positive results, otherwise patients were reported as *F*. *nucleatum* negative if both tests yielded negetive results. Table 1 shows the baseline characteristics of the study subjects. Histological assessment was performed according to the updated Sydney classification by three pathologists. The study was approved by the Institutional Review Board at Army Medical University, and all patients signed informed consent before participation. All experiments were performed in accordance with the relevant guidelines and regulations.

**Table 1:** Clinical characteristics of CRC patients with or without *F*. *nucleatum* infection.

Characteristic	CRC (n = 15)			Control (n = 10)	
	Early-stage *Fn*+ (n = 5)	Advanced-stage *Fn+* (n = 5)	*Fn-* (n = 5)	*Fn+* (n = 5)	*Fn*- (n = 5)
Age (median [range])	40 [23–57]	42 [24–60]	42 [24–60]	41 [26–56]	41 [27–55]
Sex					
Male	2	2	3	2	3
Female	3	3	2	3	2
Site					
Colon	5	5	5	5	5
Rectal	0	0	0	0	0
AJCC stage					
0	1	0	0	0	0
I	4	0	1	0	0
II	0	0	3	0	0
III	0	2	1	0	0
IV	0	3	0	0	0
Smoker	1	2	1	1	1
Drinker	0	1	1	1	0

*Fn* = *Fusobacterium nucleatum*; CRC = colorectal cancer; control = paracancerous tissues; *Fn*+ = current active *F*. *nucleatum* infection by both bacterial culturing and 16S rRNA tests; *Fn*- = the absence of *F*. *nucleatum* by both bacterial culturing and 16S rRNA tests.

### RNA isolation and microarray analysis

RNA was extracted from CRC tissues and their adjacent normal tissues using TRIzol (Invitrogen, #15596026) and an miRNeasy Mini Kit (Qiagen, #217004) according to the manufacturer’s instructions. The RNA yield and purity were determined using a NanoDrop ND-1000 spectrometer, and RNA integrity was assessed by electrophoresis. Only RNA samples with sufficient integrity and OD260/280 ratios indicating high-quality RNA were included in microarray analysis. The expression profiles of miRNAs and mRNAs were dertermined using an Affymetrix GeneChip miRNA 3.0 Array and a GeneChip Human Transcriptome Array 2.0. The results of the miRNA and transcriptome arrays have been deposited in Gene Expression Omnibus (GEO) database of the National Center for Biotechnology Information (NCBI) with accession codes GSE122182 and GSE122183, respectively.

### Cell culture and *F*. *nucleatum* identification

The human epithelial colorectal cell line Caco-2 was provided by the cell bank of the Chinese Academy of Sciences. Caco-2 or HEK-293 human embryonic kidney cells were cultured in DMEM (Gibco, 11965–092) or RPMI 1640 medium (Gibco, 11875–093) supplemented with 100 U/mL streptomycin/penicillin (Gibco,15140–122) and 10% fetal bovine serum (FBS, Gibco,10099–141) at 37°C in an atmosphere containing 5% CO_2_ and 95% air.

The specimens were spread onto Tryptic Soy Broth plates containing 5% defibrinated sheep blood at 37°C for 2 d under anaerobic conditions (10% H_2_, 5% CO_2_, and 85% N_2_) using the Anoxomat MarkII anaerobic gas filling system (Mart Microbiology, Netherland), and single colony was selected for bacterial identification.

### Real-time RT-PCR

The expression of miR-4474/4717 or CREBBP mRNA from CRC tissues and their adjacent normal tissues was evaluated using TaqMan miRNA assays (Ambion, #4440886) or PrimeScript RT-PCR kits (Takara, DRR037) with an ABI 7500 real-time PCR system (Applied Biosystems, SA). The reaction parameters were as follows: 94°C for 2 min, followed by 39 cycles of 94°C for 15 s and 60°C for 30 s. The expression of the housekeeping genes β-actin or RNU6-1 was used as endogenous control to normalize the expression of mRNA or miRNAs. The sequences of the primers used were as follows: CREBBP, forward-5’-CGTGTCACAGGGACAGGTG-3’, and reverse-5’-GTGACTGTGTCACTGGAGGG-3’, and β-actin forward-5’-TTCCTTCCTGGGCATGGAGTCC-3’, and reverse-5’-TGGCGTACAGGTCTTTGCGG-3’. The expression of each miRNA relative to that of the RNU6-1 or β-actin genes was calculated using the comparative CT (ΔΔCT) method.

### Western blot analysis

Western blot analysis was performed by following standard procedures reported elsewhere [14]. Antibodies against CREBBP(CST, 7389) and β-actin (CST, 4967) were obtained by the Cell Signaling Technology.

### Plasmid construction and luciferase assay experiments

The pMIR-REPORT luciferase vector (Ambion, AM5795) was used to generate the miR-4474/4717 target CREBBP construct according to the manufacturer’s instructions. The 3’-untranslated region (3’UTR) of CREBBP (164 or 96 bp) was amplified using cDNA from Caco-2 cells using the following primers: miR-4474, forward-5’-CAGTGAGAAAGGTCCCCCAC-3’, and reverse-5’-GGGATGAACTTCAGCTCCCC-3’; and miR-4717, forward-5’-GCCTGCTGGGTTCTTAACCT-3’, and reverse-5’-GGCTCTAGCCCCACTTCTTG-3’. Another construct containing a mutated binding site was also generated as a control (AGCCACA to CATATC or CCATGTG to ATGATCT). The amplified cDNA was digested with SpeI (Takara, D1086) and *Hind*III (Takara, D1060) and then ligated into the multiple cloning site of the pMIR-REPORT luciferase vector, with the resulting constructs named pMIR-CREBBP-wt1/2 and pMIR-CREBBP-mut1/2.

HEK-293 cells were cotransfected with 0.8 μg of pMIR-CREBBP-wt or pMIR-CREBBP-mut, 0.04 μg of the Renilla luciferase control vector, and 100 nM miR-4474/miR-4717 negative control (Invitrogen, 4464058), mimic (Invitrogen, 4464066), or inhibitor (Invitrogen, 4464084) with Lipofectamine 2000 for 24 h. miR-4474/miR-4717 inhibitor are small, chemically modified single-stranded RNA molecules designed to specifically bind to and inhibit endogenous miRNA molecules and enable miRNA functional analysis by down-regulation of miRNA activity. The luciferase activities were subsequently evaluated using the dual luciferase reporter assay system (Promega, E2920), and the relative activities of firefly luciferase were normalized against that of the Renilla luciferase.

### Gene ontology (GO) and pathway analysis

A gene ontology (GO) analysis was performed to assign genes to different GO terms according to their annotations [15]. The biological function of genes can be better understood via integrated analysis of the Kyoto Encyclopedia of Genes and Genomes (KEGG), Biocarta and Reactome Pathways[16,17]. The significance of the enrichments were reflected by the P-values and were evaluated using the false discovery rate (FDR). The significant GO terms and pathways were filtered with the criteria of *P*< 0.001 and FDR < 0.05.

### Statistical analysis

The random-variance model (RVM) t-test or F-test was used to filter out the differentially expressed genes for the control and experiment groups [18]. Multiple testing correction was employed for microarray clustering analysis. Pairwise and multiple comparisons were performed using Student’s t-test and one-way ANOVA, respectively. All statistical analyses were performed using SPSS version 13.0.

## Results

### miR-4474 and miR-4717 are associated with *F*. *nucleatum*-induced CRC

**Fig 1:**
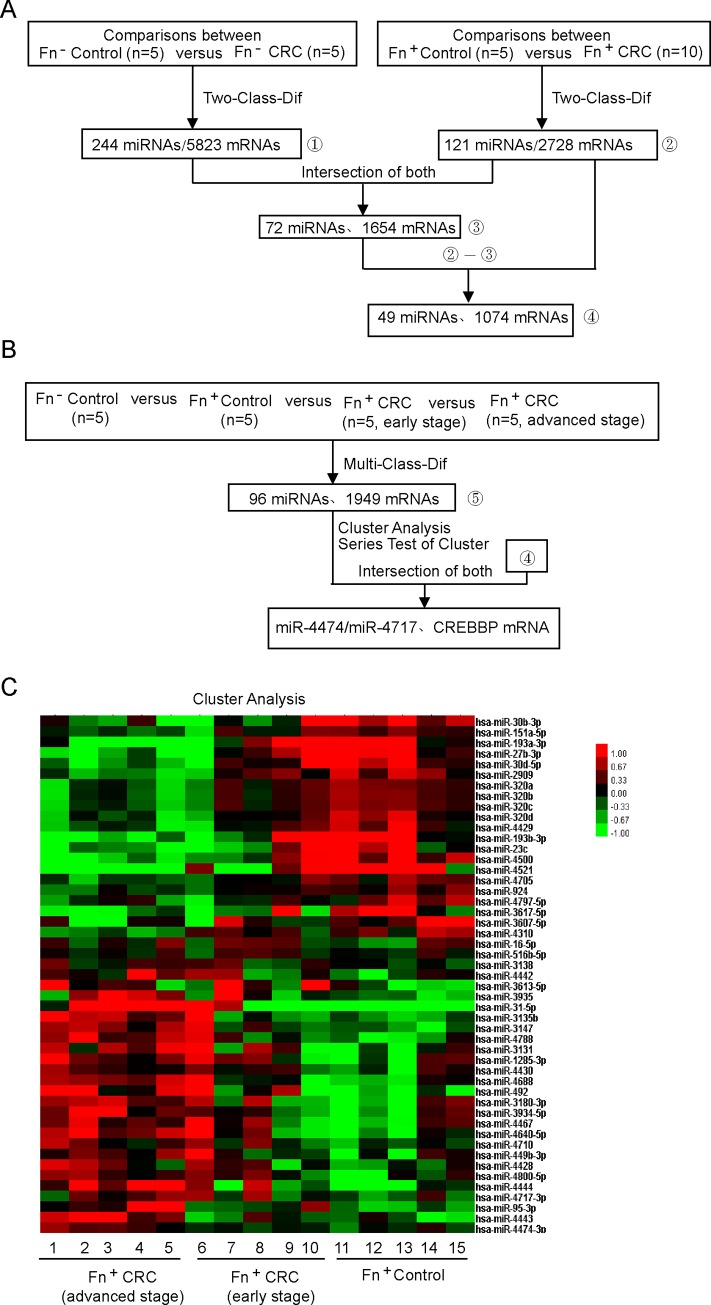
MiR-4474 and miR-4717, which were screened and identified as being associated with *F*. *nucleatum*-induced CRC. (A and B) Schematic flow of the study. Fn = *Fusobacterium nucleatum*; control = paracancerous tissues; CRC = colorectal cancer. Total RNA from CRC and adjacent normal tissues with or without *F*. *nucleatum* infection were used to perform the microarray assay. (C) The down- and upregulated miRNAs in hierarchical clustering analysis. Two subclasses, *F*. *nucleatum*-positive CRC (n = 10) and *F*. *nucleatum* positive control (n = 5), exhibited clustering results of 49 miRNAs with 2-fold changes in *F*. *nucleatum*-induced CRC.

To identify potential miRNAs involved in *F*. *nucleatum*-induced CRC, we examined the miRNA and mRNA expression profiles in CRC tissues and their adjacent normal tissues (control group) with or without *F*. *nucleatum* infection through microarray analysis. Through a Two-Class-Dif analysis, 244 miRNAs were identified as being aberrantly expressed in *F*. *nucleatum*-negative CRC tissue compared with the *F*. *nucleatum*-negative control, while 121 miRNAs were aberrantly expressed in *F*. *nucleatum*-positive CRC tissue compared with the *F*. *nucleatum* positive control. Among these miRNAs, 72 miRNAs were identified from both comparisons. Thus, the expression of 49 miRNAs involved in *F*. *nucleatum-*induced CRC varied by at least 2-fold and differed significantly between the *F*. *nucleatum*-positive CRC and *F*. *nucleatum* negative CRC (Fig 1A and 1C). In a further screen to identify miNRAs associated with *F*. *nucleatum*-positive CRC, 96 miRNAs were identified as being significantly differentially expressed in early and advanced stage of CRC testing positive for *F*. *nucleatum* infection through a Multi-Class-Dif analysis (Fig 1B). In addition to the results of this analysis, miR-4474 and miR-4717 were identified through screening as being associated with *F*. *nucleatum*-induced CRC in the following validation assay.

### CREBBP is the primary aberrantly expressed gene in *F*. *nucleatum*-induced CRC

**Fig 2:**
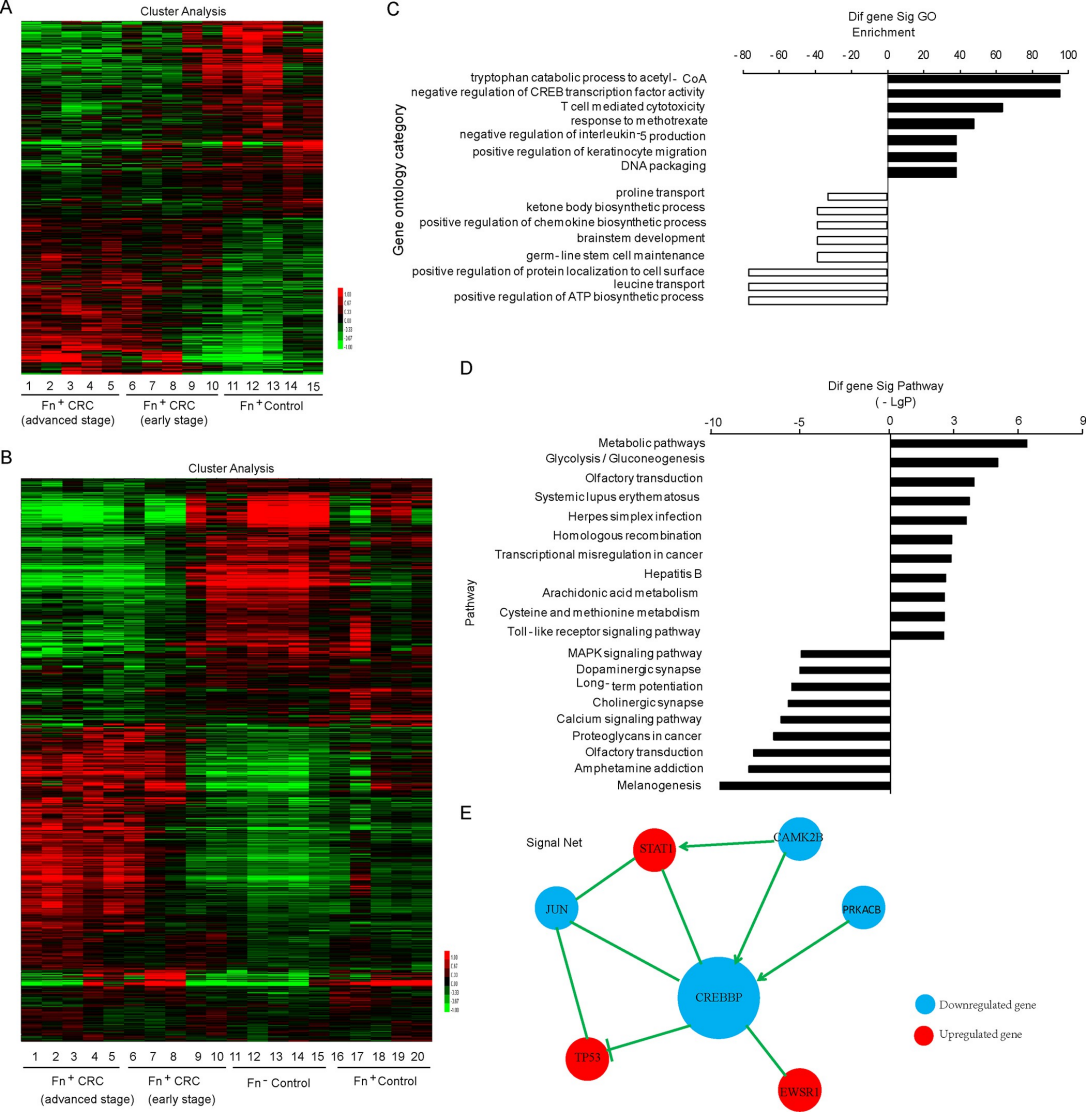
CREBBP is the primary aberrantly expressed gene in *F*. *nucleatum*-induced CRC. (A and B) Hierarchical clustering analysis of aberrantly expressed genes in *F*. *nucleatum*-induced CRC. Up- and downregulated genes are shown in red and green, respectively. The fold changes in mRNA expression in *F*. *nucleatum*-positive CRC versus the *F*. *nucleatum* positive control. (C) GO enrichment analysis of aberrantly expressed mRNAs. The vertical axis represents the pathway category, and the horizontal axis represents the enrichment of pathways. The most enriched GO pathways were screened in accordance with *P*<0.001 and FDR<0.05. (D) KEGG pathway analysis of aberrantly expressed mRNAs. The most enriched pathways were filtered by the criteria of *P*< 0.001 and FDR < 0.05. (E) The protein-protein interaction network of the major aberrantly expressed genes was generated. Up- and downregulated genes are shown in red and blue, respectively.

According to the Two-Class-Dif analysis, the expression of 1074 mRNAs involved in *F*. *nucleatum-*induced CRC were significantly altered by at least 2-fold in the *F*. *nucleatum-*positive CRC and the positive control tissues (Fig 2A). The expression of 1949 mRNAs were observed to be significantly altered between the early and advanced stage CRC with *F*. *nucleatum* positive infection by the Multi-Class-Dif analysis (Fig 2B). GOs were significantly regulated by *F*. *nucleatum* (*P*<0.001 and FDR<0.05). The upregulated GOs included tryptophan catabolism to acetyl-CoA, the negative regulation of CREB transcription factor activity, T cell mediated cytotoxicity, the response to methotrexate, and the negative regulation of interleukin-5 production. In contrast, significantly downregulated GOs included the positive regulation of the ATP biosynthetic process, leucine transport, the positive regulation of protein localization to the cell surface, the apoptotic signaling pathway, and the chemokine biosynthetic process. All of the GOs that were highly enriched are shown in Fig 2C. The KEGG analysis results indicated that several signaling pathways were either up- or downregulated in *F*. *nucleatum*-induced CRC tissue that involved cellular processes such as metabolic pathways, glycolysis/gluconeogenesis, calcium signaling, transcriptional dysregulation in cancer and pathways in cancer. Fig 2D shows the top 40 and 15% of the up- and downregulated signaling pathways of highly enriched GOs, respectively. Additionally, the signaling network of overlapping genes was selected in the GO and pathway analysis was generated (Fig 2E). The primary aberrantly expressed genes are shown according to the network, including CREBBP, JUN, PRKACB, CAMK2B, STAT1, TP53 and EWSR1.

### Expression of miR-4474/4717 and CREBBP mRNA by real-time RT-PCR analysis

**Fig 3:**
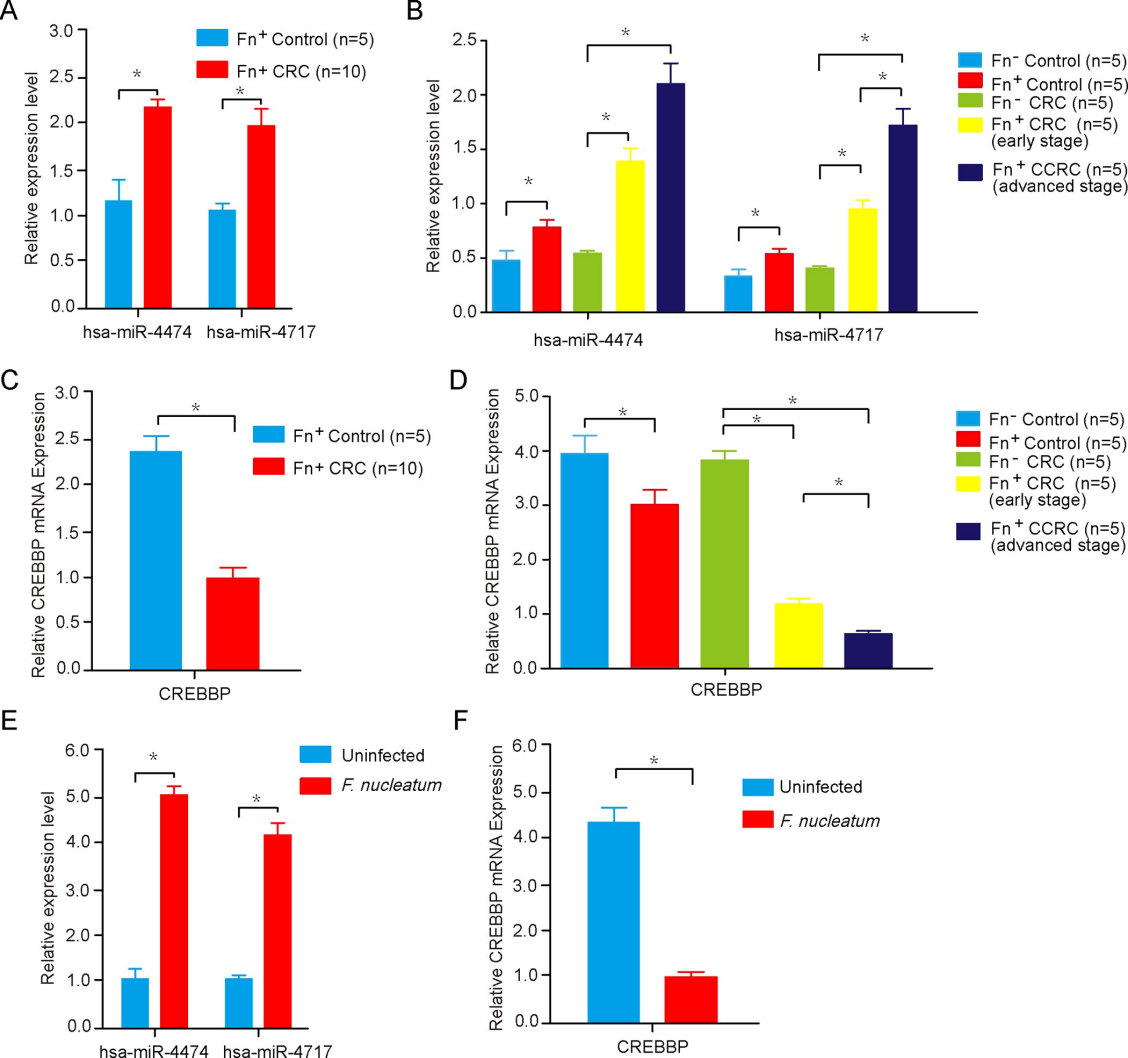
Expression of miR-4474/4717 and CREBBP mRNA by real-time RT-PCR analysis. (A and B) The expression of miR-4474/4717 was detected by real-time RT-PCR in 15 CRC patients and 10 paracancerous tissues with or without *F*. *nucleatum* infection. (C and D) The expression of CREBBP mRNA was detected by real-time RT-PCR in clinical specimens. (E and F) The expression of CREBBP mRNA was detected by real-time RT-PCR in Caco-2 cells after *F*. *nucleatum* infection 24 hours. The results shown are representative of at least three independent experiments. *P<0.05.

To verify the microarray data, the expression of miR-4474/4717 was determined by real-time RT-PCR in colorectal tissues obtained from CRC patients and paracancerous normal tissues infected with *F*. *nucleatum*. As shown in Fig 3A and 3B, the expression of miR-4474/4717 in *F*. *nucleatum-*positive CRC tissues was significantly higher than that observed in the *F*. *nucleatum* positive controls and was also significant in the early- and advanced-stage CRC with *F*. *nucleatum* positive infection (*P*<0.05). Furthermore, the expression levels of miR-4474/4717 in advanced-stage CRC with *F*. *nucleatum* positive infection was more than the early-stage ones (Fig 3B). However, the expression of CREBBP mRNA was significantly lower in *F*. *nucleatum-*positive CRC tissues than in the *F*. *nucleatum* positive controls (*P*<0.05) (Fig 3C) and was also lower in the early- and advanced-stage CRC with *F*. *nucleatum* positive infection (*P*<0.05) (Fig 3D). Interestingly, the expression levels of CREBBP mRNA in advanced-stage CRC with *F*. *nucleatum* positive infection was lower than the early-stage ones (Fig 3D). In agreement with colorectal tissues findings, a significant increase in miRNA-4474/4717 expression was observed in Caco-2 cells infected with *F*. *nucleatum* (*P*<0.05 vs. uninfected)(Fig 3E), while the expression of CREBBP was decreased in *F*. *nucleatum* infection (*P*<0.05 vs. uninfected)(Fig 3F). The quantitative RT-PCR results demonstrated that the expression of miR-4474/4717 and CREBBP mRNA was up- or downregulated in *F*. *nucleatum-*positive CRC tissues and gradually increased or decreased as a result of the malignancy of CRC, respectively.

### CREBBP is a novel target of miR-4474/4717

**Fig 4:**
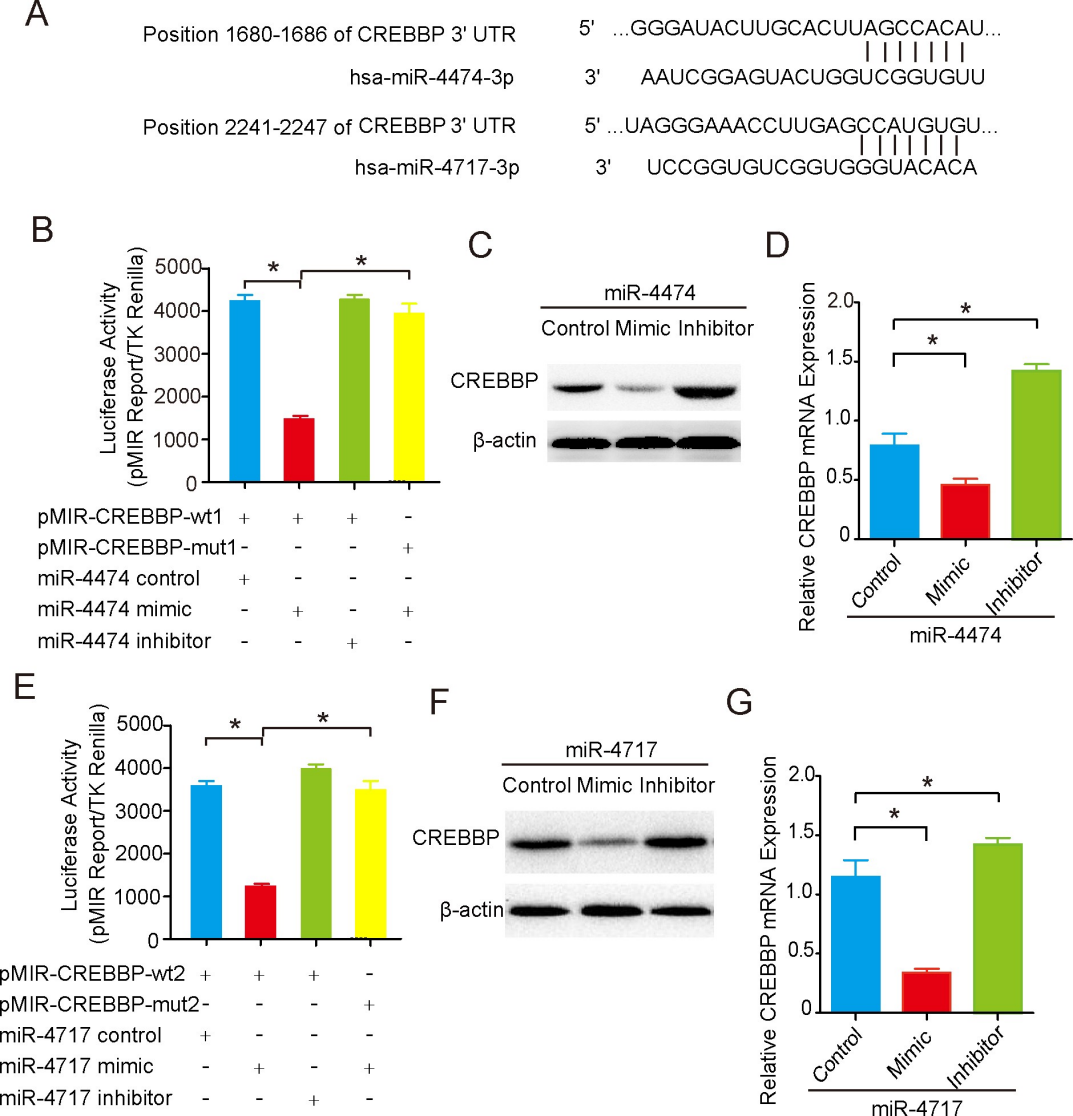
CREBBP is a novel target of miR-4474/4717. (A) The sequences of miR-4474/4717 and the potential binding site in the 3’UTR of CREBBP. (B and E) Luciferase reporter assay. HEK293 cells were transiently cotransfected with 0.8 μg of pMIR-CREBBP-wt or pMIR-CREBBP-mut, 0.04 μg of Renilla luciferase control vector, and 100 nM miR-4474/miR-4717 control, mimic, or inhibitor with Lipofectamine 2000 for 24 h. (C,F, D and G) Western blot and qRT-PCR analyses. Human epithelial colorectal Caco-2 cells were transfected with miR-4474/4717 control, mimic or inhibitor for 24 h, and the mRNA and protein expression of CREBBP was detected by qRT-PCR and Western blot analyses. The results shown are representative of at least three independent experiments. *P< 0.05.

Using TargetScan Human 7.2 and miRanda, CREBBP was identified as a putative miR-4474/4717 target gene (Fig 4A). To assess whether CREBBP is a target of miR-4474/4717, vectors containing the wild-type (pMIR-CREBBP-wt1/2) or mutant 3’UTR (pMIR-CREBBP-mut1/2) of CREBBP mRNA were generated by individually fusing the UTR constructs downstream of the firefly luciferase gene. As shown in Fig 4B and 4E, the relative luciferase activity of the wild-type vectors was effectively suppressed by the two miRNA mimics, while that of mutant vectors was not, suggesting that miR-4474 and miR-4717 can bind to the 3’UTR of CREBBP to downregulate its expression (*P*<0.05). To verify the results, miR4474/4717 mimics were transfected into Caco-2 cells, which resulted in the reduced expression of CREBBP protein (Fig 4C and 4F). In addition, the mRNA levels of CREBBP also showed a 50 or 80% reduction in the presence of miR4474/4717 mimics (*P*<0.05) (Fig 4D and 4G). These results suggest that CREBBP is a novel target of miR-4474/4717, which can decrease the expression of CREBBP via mRNA degradation.

## Discussion

Based on the results of this study, miR-4474/4717 and CREBBP are proposed to play important roles in *F*. *nucleatum-*induced CRC. First, miR-4474 and miR-4717 were identified in a screen as being associated with *F*. *nucleatum*-induced CRC. Subsequently, bioinformatics analysis identified CREBBP as the primary aberrantly expressed gene in *F*. *nucleatum*-induced CRC tissue. In addition, the expression of miR-4474/4717 and CREBBP mRNA was demonstrated by real-time RT-PCR analysis, and CREBBP was identified as a novel target of miR-4474/4717. Thus, the results of this study provides new insights into the mechanism of *F*. *nucleatum*-induced CRC.

MiRNAs are regulators of gene expression and have been shown to be involved in the regulation of various pathways, including cancer, development, differentiation, signal transduction and cell maintenance [19,20]. Oncogenic miRNAs (miR-17-92a and miR-25-106b clusters) have been shown to be functionally involved in CRC progression [5]. Additionally, various miRNAs, including miR-21, miR-96, miR-135, miR-31, miR-224, miR-200c and miR-155 have been shown to be associated with the pathogenesis of CRC [9]. However, few studies have investigated miRNA expression with respect to *F*. *nucleatum*-induced CRC. Therefore, we evaluated the expression profile of miRNAs using CRC tissues and paracancerous normal tissues with or without *F*. *nucleatum* infection using an Affymetrix miRNAs array platform. The results of the microarray analysis indicated that 49 miRNAs are involved in *F*. *nucleatum-*induced CRC, and 96 miRNAs were significantly differentially expressed in early and advanced stage CRC with *F*. *nucleatum* positive infection by a Multi-Class-Dif analysis. Among these differentially expressed miRNAs, the expression of miR-4474 and miR-4717 were upregulated in CRC with *F*. *nucleatum* positive infection compared with the *F*. *nucleatum* positive control. In addition, the results of a real time RT-PCR analysis further confirmed the overexpression of miR-4474 and miR-4717 in *F*. *nucleatum-*induced CRC. To the best of our knowledge, this is the first time that miR-4474 and miR-4717 have been shown to be involved in *F*. *nucleatum-*induced CRC.

In this study, we demonstrated that the expression of CREBBP mRNA was correlated with CRC metastasis in a set of colorectal carcinomas with or without *F*. *nucleatum* infection. The differentially expression of CREBBP mRNA was identified, validated and subjected to GOs and KEGG analysis. Highly enriched GOs regulated by *F*. *nucleatum* infection may involve biological processes such as tryptophan catabolism to acetyl-CoA, the negative regulation of CREB transcription factor activity, T cell mediated cytotoxicity, the response to methotrexate, and the negative regulation of interleukin-5 production. According to the KEGG analysis results, specific pathways targeted by miR-4474/4717 were identified as being involved in the progression of *F*. *nucleatum-*induced CRC. The signaling network of overlapping genes was selected in GOs and the pathway analysis results identified the primary aberrantly expressed genes, including CREBBP, JUN, PRKACB, CAMK2B, STAT1, TP53 and EWSR1. Additionally, CREBBP was demonstrated to be a novel target of miR-4474/4717. Thus, the results suggest that the progression of *F*. *nucleatum-*induced CRC may be attributed to the decreased expression of CREBBP. Consistent with this finding, many studies have shown that CREBBP affects colonic tumorigenesis and stem cell pluripotency [6,10].

In summary, the results of this study suggest the involvement of miR-4474/4717 in the progression of *F*. *nucleatum-*induced CRC through the posttranscriptional regulation of CREBBP. Nevertheless, there are several limitations of this study. First, the microarray experiments only assayed 15 CRC and 10 paracancerous tissues, and the role and mechanism of miR-4474/4717 in the progression of *F*. *nucleatum-*induced CRC has not been fully elucidated. Future studies will focus on determining the distinct role and mechanism of these aberrant miRNAs by using additional clinical cases.
